# Successional Trajectories of Deep Subsurface Microbiomes in Response To Experimental Dihydrogen Injection

**DOI:** 10.1007/s00248-026-02697-3

**Published:** 2026-02-11

**Authors:** Antoine Lafont, Cyrille Violle, Magali Ranchou-Peyruse, Marion Guignard, Jean Mura, Tiphaine Fargetton, Pierre Cézac, Anthony Ranchou-Peyruse

**Affiliations:** 1 Universite de Pau et des Pays de l’Adour, E2S UPPA, CNRS, IPREM, Pau, France; 2 CEFE, Univ Montpellier, CNRS, EPHE, IRD, Montpellier, France; 3 Universite de Pau et des Pays de l’Adour, E2S UPPA, LaTEP, Pau, France; 4 Storengy (groupe Engie), La Garenne-Colombes, France

**Keywords:** Dihydrogen, UGS, Lithotrophy, Autotrophy, Sulfate reducing bacteria, Methanogenic archaea

## Abstract

Converting pre-existing gas storage facilities to dihydrogen storage raises critical questions about storage quality and dihydrogen consumption by prokaryotes. To investigate biologically driven changes during such transitions, we analyzed data from five dihydrogen pulse experiments conducted in pressurized bioreactors that replicate deep aquifer pressure and temperature conditions. Our goal was to determine whether consistent community-level responses to dihydrogen injection could be identified. We found that dihydrogen exposure consistently led to a decline in fermentative ASVs, likely driven by environmental filtering. Hydrogenotrophic sulfate reducers initially dominated in some experiments, with total sulfate depletion observed in certain cases, followed by the emergence of methanogenic archaea. In some instances, a succession pattern involving *Thermodesulfovibrio* and *Methanothermobacter* appeared across taxonomically distinct communities, suggesting deterministic ecological processes. Additionally, we observed potential dispersal limitation and selection pressures, possibly linked to pH shifts caused by autotrophy. These findings underscore the importance of considering microbial dynamics in dihydrogen storage strategies in deep aquifers and suggest that, despite initial variability, predictable ecological succession may occur under specific geochemical conditions.

**Supplementary Information:**

The online version contains supplementary material available at 10.1007/s00248-026-02697-3.

## Highlights

Most of the time, the arrival of dihydrogen leads to a decline in diversity among microbial communities from deep environements.

Depending on sulfate concentration, two distinct community dynamics scenarios emerge.

The introduction of new nutrients in these environments stimulates microbial communities but does not alter their structure.

**Supplementary Information:**

The online version contains supplementary material available at 10.1007/s00248-026-02697-3.

## Introduction

In 1995, Stevens and McKinley were the first to theorize the existence of microbial ecosystems sustained by geochemically derived dihydrogen (H_2_; Stevens and McKinley 1995). The discovery of subsurface lithoautotrophic microbial ecosystems (SliMEs) has revolutionized our understanding of life beyond the traditionnal photosynthetic dependencies. Numerous studies have substantiated the presence of SLiMEs in deep oceanic and continental ecosystems, expanding our knowledge of microbial adaptability (Fry et al. 1997; Kotelnikova et al., 1997, Chapelle et al. 2002; Haveman et al., 2002, Takai et al. 2003; Lin et al. 2005; Basso et al. 2009; Crespo-Medina et al. 2014; Suzuki et al. 2024).

Central to these ecosystems is H_2_, a highly energetic electron donor harnessed by lithoautotrophic microorganisms, particularly when sulfate or carbon dioxide is present. Many procaryotes consume H_2_ through metabolic pathways such as methanogenesis, sulfate reduction, and acetogenesis, and produce it via fermentation, anaerobic methane oxidation, and nitrogen fixation (Gregory et al. 2019).

In natural systems, H_2_ originates mainly from geochemical processes occurring under extreme conditions for life (serpentinization, degassing, water radiolysis) (Charlou et al. 2002; Deville and Prinzhofer 2016; Murray et al. 2020; Truche et al. 2021; Lollar et al. 2014; Gaillard et al. 2011; Zgonnik 2020; Mahlstedt et al. 2022). With its minimal molecular size, H_2_ exhibits high mobility, potentially migrating from deep origins to regions more suitable for life, particularly in terms of temperature. During this ascent, H_2_ can react with various minerals, follow preferential pathways and fracture zones, and become a necessary but limiting resource for microbial groups dependent on its presence in oligotrophic conditions (Zgonnik 2020; Greening et al. 2022).

While H_2_ is an essential resource for maintaining SLiMEs, its abundance could lead to physiological constraints and inhibition of present communities (Braun and Gottschalk 1981; Morinaga and Kawada 1990; Kantzow and Weuster-Botz 2016; Jensen et al. 2019). Interestingly, artificial analogs, so-called “Artificial-SLiMEs” (Ranchou-Peyruse et al. 2024a), of these subsurface conditions have been created and may become increasingly widespread in the coming years. Between the 1950s and 1990s, large quantities of manufactured town gas, containing about 50 to 60% H_2_, were stored in deep aquifers in Europe (France, Germany, Czechia) (Pankhurst 1967; Foh et al. 1979; Taylor et al., ; Šmigáň et al. 1990; Stolten and Emonts 2016; Ebrahimiyekta 2017). A decrease in the concentration of H_2_, CO_2_, and CO, as well as an increase in sulfides and methane, were noted in some of the reservoirs and, in the case of the Lobodice storage (Czechia), linked to microbial activities. These underground gas storage (UGS) facilities gradually evolved from storing town gas to natural gas, primarily composed of methane. Today, numerous studies highlight the need to develop massive H_2_ storage (i.e., UGS in salt caverns, depleted reservoirs, and deep aquifers) to prepare for its future deployment, in line with energy policies concerned with its role in climate change mitigation. Some pilot projects have begun injecting H_2_ into depleted hydrocarbon reservoirs, resulting in methane production through methanogenesis and the formation of Artificial-SLiMEs (Ehinger et al. 2009; Tyne et al. 2021; Thaysen et al. 2023; Hellerschmied et al. 2024; Ranchou-Peyruse et al. 2024a; Wang et al. 2024). Moreover, experimental simulations of H_2_ injection into porous reservoirs have been conducted in high-pressure reactors (Haddad et al. 2022a; Dohrmann and Krüger 2023; Hellerschmied et al. 2024; Mura et al. 2024a, b). Some of these experiments were performed in triphasic reactors reproducing the aquifer rock, formation water with indigenous microorganisms, and a gas phase simulating injected natural gas. These microbial ecosystems provide interesting simplified models to assess the effect of H_2_ on microbial communities. The estimated water flow rate within the formation is on the order of a few meters per year (Ranchou-Peyruse et al. 2019), with artificial acceleration near the storage site due to gas injection and extraction. From an energy metabolism perspective, the essential microbial groups are divided among fermentative microorganisms, sulfate-reducing bacteria, and those that respire CO_2_ (i.e., methanogens and acetogens). Although sampling requires specific procedures (Basso et al. 2009; Ranchou-Peyruse et al. 2023), UGS in deep aquifers present an opportunity to study the microbiology of these unique geological structures. These experiments demonstrated a significant impact of microbial activity on the water’s physicochemistry and the evolution of gas and solid phases. These laboratory and in situ experiments are essential for generating data to numerically simulate these reservoirs, assess their sustainability, and evaluate their H_2_ consumption yield, which is indispensable for developing future numerical biogeomodels (Tremosa et al. 2023; Wu et al. 2024; Zeng et al. 2024). These closed-system experiments also serve as ideals models for closely evaluating the impact of H_2_ as an environmental forcing variable on the structuring of deep microbial communities. In five of these experiments, phylogenetic data based on high-throughput sequencing of *16 S rRNA* genes were generated as part of multidisciplinary studies aimed at assessing the overall impact of a sudden and significant influx of H_2_ into UGS in deep aquifers.

This study highlights H_2_ as a crucial environmental variable influencing the structuring of subsurface microbial communities in oligotrophic deep ecosystems that have not previously been exposed to H_2_. We hypothesize that H_2_ availability may selectively inhibit certain microorganisms while promoting lithoautotrophic species, potentially driving deterministic mechanisms that lead to convergent evolution within these subsurface communities influenced by H_2_. Through these studies, we aim to deepen our understanding of the ecological dynamics at play and explore the potential applications of this knowledge in sustainable energy storage solutions in porous reservoirs.

## Materials and Methods

### Origin of the Sample

Five UGS in deep aquifers belonging to different geological formations were studied, with sampled waters collected from depths ranging from 582 to 1185 m above mean sea level (AMSL), under pressures of 60 to 115 bar, and temperatures between 35 and 53 °C (Table 1). Some sites can thus be classified as mesothermic (Aquifers 2, 3, and 4), while others are hyperthermic (Aquifers 1 and 5). Regardless of the geological layer, depth, or temperature, different sulfate concentrations were measured in the sampled waters: 9 mg L^− 1^ (Aquifer 4), 14 mg L^− 1^ (Aquifer 1), 951 mg L^− 1^ (Aquifer 5), 1,252 mg L^− 1^ (Aquifer 2), and 2,407 mg L^− 1^ (Aquifer 3). The sampled wells are located in the Parisian Basin (France), except for well Ab_L_1 (Aquifer 4 in this study), which comes from the southern Aquitaine Basin (Ranchou-Peyruse et al. 2019). The wells used and the sampling periods allowed water to be sampled at the interface of the presently stored natural gas. All samplings are described in the associated publications (Haddad et al. 2022a, b; Mura et al. 2024a, b, 2025). The full set of formation water physicochemical conditions is compiled in the supplemental material (Table S1). Briefly, formation water containing indigenous microorganisms was collected on-site using a specially prepared downhole sampler to ensure strictly aseptic conditions (Ranchou-Peyruse et al. 2023). Two samplers each collected 600 mL of formation water and were brought to the surface while maintaining bottom-hole pressure throughout the procedure. Upon arrival at the laboratory, the sampled water was carefully depressurized (< 1 bar·min^− 1^) and stored anoxically at 4 °C in vials until use in the high-pressure reactor.

**Table 1:** Main physico-chemical characteristics of the studied and simulated sites (from Haddad et al. 2022a; Mura et al. 2024a, b,^2025^). Depth unit is meters above mean seal level (AMSL). *Sulfate concentrations are expressed at the beginning of the experiment

Well names	Geological strata	Depth (AMSL)	Temperature (°C)	Pressure (bar)	Sulfate (mg L^− 1^)*	Number of H_2_ injection	% of H_2_ in the gas phase	Solubility of H_2_ in water during the first injection (mmol H_2_∙Kg of water^− 1^)	References
Pb_T_1 (Experiment 1)	Triassic (Rhaetian-Norian) -245 to -205 My	989	47	95	14	1	10	6.66	Haddad et al. 2022a
Pb_J_11 (Experiment 2)	Jurassic (Sequanian) -154 to -135 My	836	35	85	1252	1	10	5.09	Mura et al. 2024a
Pb_C_5 (Experiment 3)	Cretaceous (Neocomian) -96 to -108 My	840	41	85	2407	3	2	1.06	Mura et al. 2024a
AB_L_1 (Experiment 4)	Lutetian -46 to -40 My	582	36	60	9	1	2	0.95	Mura et al. 2024b
Pb_T_5 (Experiment 5)	Triassic (Rhaetian-Norian) -245 to -205 My	1185	53	115	951	3	2	0.75	Mura et al. 2025

### Experiments

Since 2020, a high-pressure, triphasic reactor (rock, formation water with native microorganisms, CH_4_/CO_2_/H_2_) has been used to simulate the injection of H_2_ into five deep aquifers serving as underground gas storage (UGS), as previously described in Haddad et al. 2022a); Mura et al. (2024a, b, 2025). These approaches, while allowing for the best possible simulation of deep environments, lack controls (abiotic, conditions without H_2_ injection) due to logistical reasons. For each experiment, the water was introduced into a sterile reactor containing a rock sample from the core of each aquifer reservoir, then pressurized and heated according to each site’s conditions (60–95 bar; 36–53 °C; see Table 1). Initially, the gas phase consisted of 99% methane (CH_4_) and 1% carbon dioxide (CO_2_), supplemented with 10 ppm benzene and 10 ppm toluene to simulate the composition of synthetic natural gas. After an incubation period necessary for the revival of sulfate-reducing activity, H_2_ was injected into the gas phase as follows: Experiments 1 and 2 received 10% H_2_, Experiments 3 and 5 had three injections up to 2% H_2_, and Experiment 4 received 2% H_2_ once. The change in concentration observed between Experiment 2 and Experiment 3 related to the shift of recommended H_2_ concentration in a storage from 10% to 2% of gas phase (Sánchez and Wasserfachs, 2023). The five experiments lasted between 87 days (Experiment 2) and 163 days (Experiment 3), during which the composition of the liquid and gaseous phases was monitored weekly using ion chromatography (Dionex Integrion HPIC, ThermoFisher Scientific) and gas chromatography (GC-mTCD; Micro GC Fusion; Chemlys; France), as described in Haddad et al. (2022a, b). At key points defined by physico-chemical monitoring, larger liquid samples (70 mL) were collected to track microbial community dynamics. To extend the duration of the experiments, formation water filter-sterilized through a PES 0.1 μm membrane filter was injected into the reactors of Experiments 1, 3, 4, and 5 on the 31st, 9th, 6th and 7th days after H_2_ injection, respectively. Microbial biomass was concentrated by filtering through PES membrane filters (47 mm diameter, 0.1 μm pore size, Sartorius Stedim). Nucleic acids were extracted using the Fast RNA ProSoli Direct Kit (MPBio) after grinding the samples in liquid nitrogen. DNA and RNA were separated using the All Prep RNA/DNA Kit (Qiagen). The RNA was reverse-transcribed using M-MLV reverse transcriptase (Invitrogen™). The V4-V5 region of the *16 S rRNA* gene from extracted DNA and cDNA was amplified using the universal primers 515 F-928R (Wang and Qian 2009). Sequencing was performed using Illumina’s MiSeq 2 × 250 bp technology at the GenoToul genomics facility in Toulouse, France. Raw sequencing data (*16 S rRNA* gene) and related metadata were obtained from the NCBI Sequence Read Archive (SRA) under the Bioprojects IDs: PRJNA813384, PRJNA1023401, and PRJNA1023500. These correspond to the experiments on Pb_T_1 (coded as Experiment 1 in this study for the Aquifer 1), Pb_J_11 (Experiment 2), Pb_C_5 (Experiment 3), Ab_L_1 (Experiment 4) and Pb_T_5 (Experiment 5) (Haddad et al. 2022a; Mura et al. 2024a, b, 2025).

### Data and Statistical Analyses

**Fig. 1:**
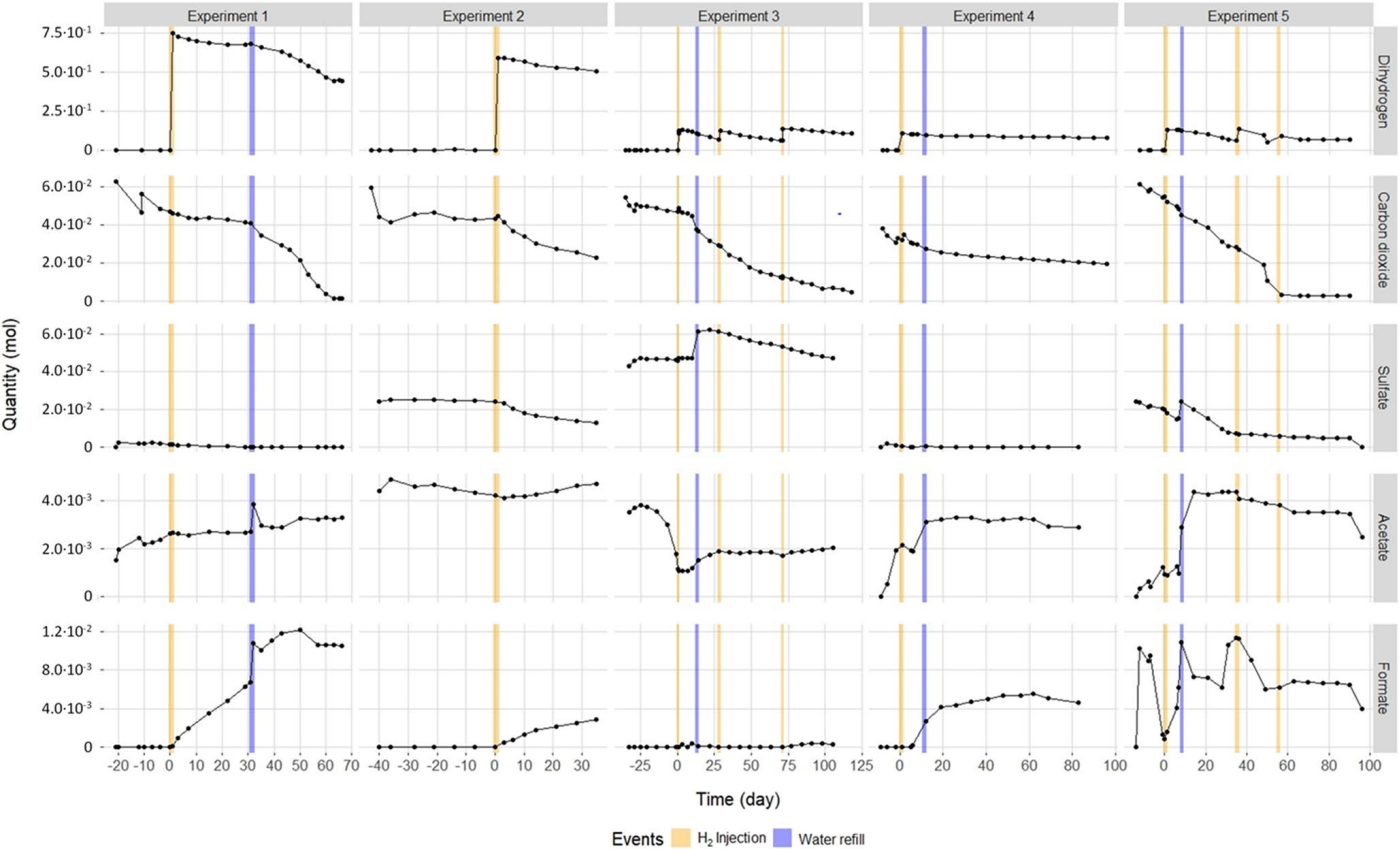
Major environmental variable dynamics in each incubation. The x-axis shows time in days, centered on H_2_ injection. The y-axis shows the molar quantity of each product, corrected for sampling loss. Columns segregate experiments, and “raw” indicates the tracked environmental variable. Dihydrogen and carbon dioxide were monitored in the gaseous phase, while sulfate, acetate, and formate were measured in the liquid phase. Orange lines represent H_2_ injection, and blue lines represent water refill

All statistical analyses were performed under R studio (2.6-4; R Core Team (2022). Experiments produced five temporal datasets containing the dynamics of the gas phase (H_2_, CO_2_, CH_4_) and the liquid phase (sulfate, acetate, formate, carbonate, fluoride, chloride, sodium, potassium, magnesium, and calcium). To illustrate biological reactivity within the system, the dynamics of H_2_, CO_2_, sulfate, hydrogen sulfide, acetate and formate dynamics are shown in Fig. 1.

### Analysis of Prokaryotic 16 S rRNA Sequences

The MiSeq sequencing data (DNA and cDNA) were processed using QIIME2 (version 2022.11, Bolyen et al. 2019). After quality analyses, the demultiplexed sequences were trimmed with DADA2 (Callahan et al. 2016). Singleton sequences were removed. Amplicon sequence variants (ASVs) were then taxonomically assigned using the Silva 138.2 database (Quast et al. 2012), and chloroplast and mitochondrial genes were excluded from the analysis.

### Alpha and Beta Diversities

**Fig. 2:**
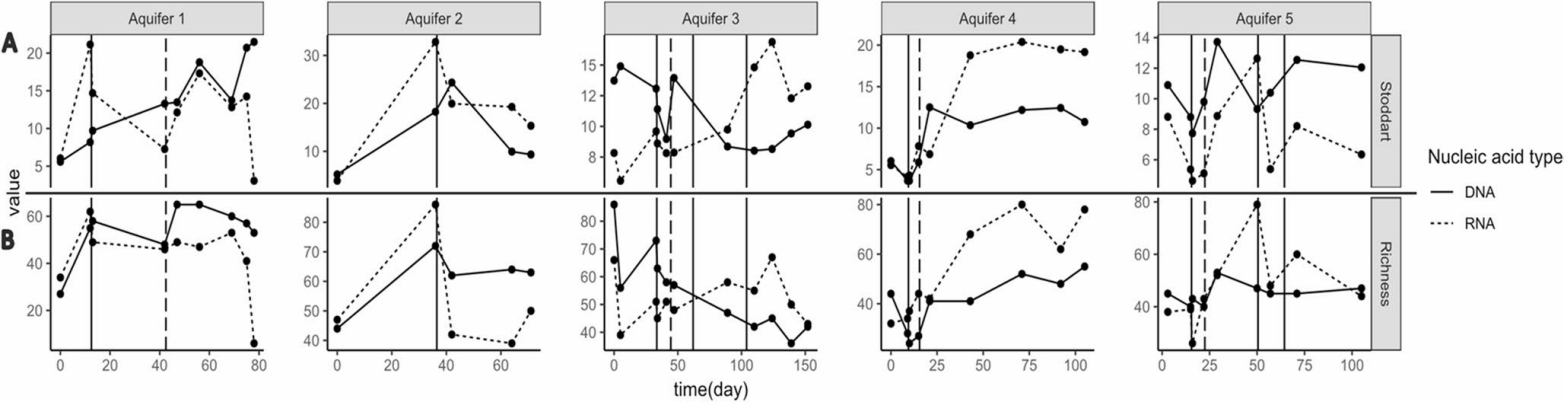
Biodiversity response of five aquifers communities to H_2_ injection. Each column represents an experiment labeled Experiment 1, Experiment 2, Experiment 3, Experiment 4, and Experimental 5. Alpha diversity is illustrated with the Stoddart index in the top row (Row A), while the bottom row (Row B) shows richness as the number of ASVs at each time point. Solid vertical lines indicate H_2_ injection into the system, and dashed lines represent water refills

In batch cultures, such as those conducted with the high-pressure reactor, inactive microorganisms (dormant states, spores), and lysed cells from necromass can persist in the closed environment. This can distort community structure when analysis is based solely on DNA data. Therefore, it is preferable to assess the dynamics of taxonomic diversity based on ASVs derived from *16 S rRNA* transcripts, as these provide a more accurate reflection of environmental changes experienced by the microbial ecosystem. Alpha diversity captures changes in overall biodiversity during incubation, indicating how H_2_ injection affects community characteristics at local scales. However, ASV structure may artificially increase richness and alter alpha diversity. To correct for this possible bias, the Stoddart index, an analog of the Simpson index, was used (Stoddart 1983; Fig. 2). Apparent ASV richness is also reported in the figure. The Shannon and Pielou indexes are provided in the supplementary materials (Pielou 1966; Figures S1). Each metric was calculated using the vegan package (version 2.6-4; Oksanen et al. 2023).

Following the observation of modifications in alpha diversity, beta diversity between experiments at corresponding time points was calculated to detect non-random patterns in community structure, indicating potential shared ASVs. Beta diversity was calculated using the Jaccard index based on a presence/absence matrices for each pair of experiments at comparable time points relative to H_2_ injection. The time points were categorized as “Before” (one day prior to injection), “After” (the first sampling after H_2_ injection), and “Furthest” (the earliest day before water addition or prior to the next H_2_ injection). These beta diversities were compared to a null distribution of 1,000 beta diversity values generated by simulating 10,000 mock communities, sampling the total biodiversity for each pair of experiments. Taxonomic beta diversities were computed using the Biodiversity Assessment Tools (BAT) package (version 2.9.6; Cardoso et al. 2015). Observed beta diversities were normalized using z-scores based on the mean and standard deviation of the null distribution. To test if wether the heterogeneity in the category “Furthest” biased the normalized z-score, we perform Spearman’s correlation test couple of sample used in beta diversity time differential and normalized beta-diversity. All test returned non significative (p-value > 5%, two-sided) (see sup.mat. Figure S2).

### Metabolic Inference Using PICRUSt2

PICRUSt2 (version 2.5.3; Douglas et al. 2020) was used to infer functional profiles by providing KEGG Orthologs (KO) on cDNA feature table. The KEGG modules selected for analysis included methanogenesis (M00567), dissimilatory sulfate reduction (M00596), and the Wood-Ljungdhal pathway (M00377). Additionally, KOs related to fermentation where carbohydrate metabolism were included, notably those significatively enriched in fermentative strains as described by Hackman and Zhang (2023). To visualize changes in the inferred metabolic profile over time, a circular network was generated. Hierarchical clustering grouped ASVs by genera and time points, and KEGG modules by KO. The circular network was created with Igraph (version 2.0.3, Csardi and Nepusz, 2006), with edge bundling performed using ggraph (version 2.2.1, Pedersen, 2016).

## Results

### Environmental Data Exploration

Prior to any H_2_ pulse, each aquifer community was incubated in a mixture of methane and carbon dioxide. The latter gradually disappeared from the gas phase in nearly all incubations, often continuously. In Experiment 2, carbon dioxide consumption began rapidly (approximately 20 mmol within a week) and then ceased until H_2_ injection. During the same period, different behaviors for acetate were observed: microorganisms from Experiments 1, 4, and 5 produced acetate; those from Experiment 3 consumed it; and acetate remained stable in Experiment 2. Additionally, formate production and consumption, as well as a sulfate consumption, occurred in Experiment 5.

Under these five experimental conditions, the solubilities of H_2_ in water during the first injection were 6.66 mmol H_2_ kg of water^− 1^ (Experiment 1), 5.09 mmol H_2_ kg of water^− 1^ (Experiment 2), 1.06 mmol H_2_ kg of water^− 1^ (Experiment 3), 0.95 mmol H_2_ kg of water^− 1^ (Experiment 4), and 0.75 mmol H_2_ kg of water^− 1^ (Experiment 5).” dans la partie résultats. After H_2_ injection, H_2_ and sulfate consumption were detected. In the case of Experiment 3, H_2_ injection did not trigger sulfate depletion. Sulfate was completely depleted in Experiment 1 (43 days after H_2_ injection), 4 (5 days after H_2_ injection), and 5 (31 days after H_2_ injection ). In Experiments 2 and 3, sulfate remained detectable in the aqueous phase. Dihydrogen injection also stimulated the production of formate in all experiments, but this remained unstable during incubation. Delayed acetate production was observed in Experiment 3 (9 days after H_2_ injection), 4 (6 days after injection), and 5 (8 days after injection). In Experiments 1 and 2, acetate levels remained stable.

Different patterns in chemical dynamics appeared concomitant with water injection. In Experiment 3, sulfate consumption was triggered on the 9th day after injection. In Experiment 5, formate disappeared and acetate appeared on the 7th days after H_2_ injection.

### Alpha Diversity

In terms of DNA, the communities from deep oligotrophic environments exhibited a relatively low richness, ranging from 27 to 45 ASVs (Fig. 2, Row B), with a maximum of 86 ASVs observed in Experiment 3. The richness measured in RNA was similar to that from DNA, within a range of 32 and 47 ASVs for all experiments, except Experiment 3, which had 66 ASVs.

Both diversity indices indicate a decrease in prokaryotic active (cDNA) taxonomic diversity after the first H_2_ injection in Experiments 1, 2, 3, and 5. The most significant loss was observed in Experiment 3, with 42 ASVs disappearing from a total of 87 ASVs just before the H_2_ injection. In contrast, the microbial community in Experiment 4 remained relatively stable in richness. In an aquifer used for energy storage as gas, the stored volume varies with injections and withdrawals, causing water movements within the reservoir rock. In these experiments, sterilized formation water was injected into simulations of Aquifers 1, 3, 4, and 5. These water influxes consistently stimulated the microbial communities and increased their diversity. Notably, in Experiment 3, water injection initiated sulfate reduction, despite the initial sulfate concentration (around 2,882 mg L^− 1^) being already sufficient, suggesting that some essential trace elements for sulfate reducers were lacking before water addition.

### Beta Diversity

**Fig. 3:**
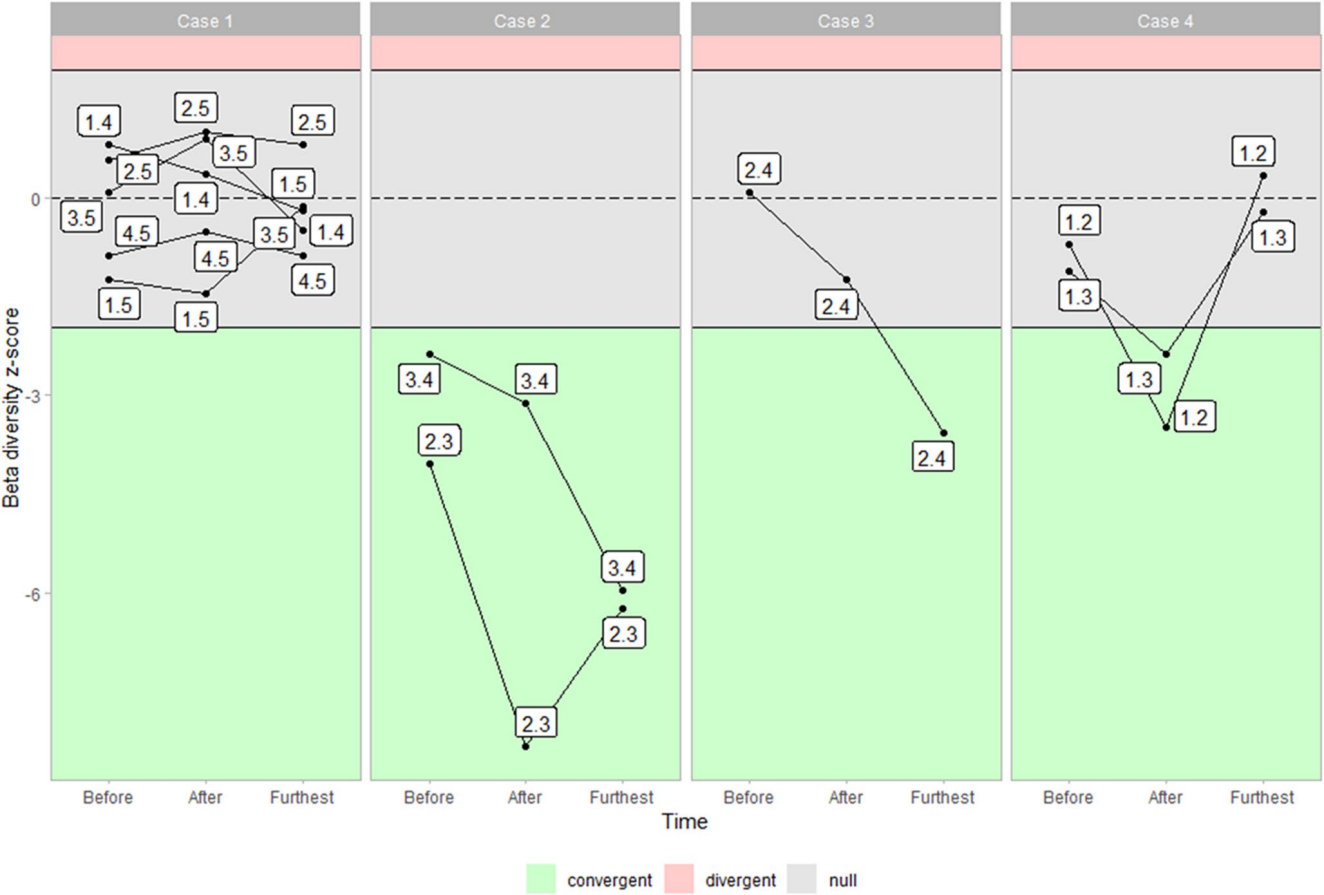
Normalized taxonomic compositional change within experiments at equivalent times after H_2_ injection. This figure shows Z-score normalized values of observed taxonomic beta diversity from cDNA data compared to neutral taxonomic beta diversity. Beta-diversity was calculated using the Jaccard distance based on the presence/absence of ASVs within the equivalent time. Neutral beta diversity was determined by creating 1,000 pairs from 10,000 mock communities formed from the ASVs of pairs of experiments, regardless of time. The time categories are: “Before” (one day prior H_2_ injection), “After” (the first sample taken after H_2_ injection), and “Furthest” (the last day after H_2_ injection without any other supplementation). Each label (e.g., “1–5”) corresponds to beta diversity between experiments 1 and 5. The dotted line represents the mean, while the solid line indicates the boundaries of the 95% quantile of the normal distribution

Before analyzing beta dissimilarity between experiments at similar times, it was noted that all normalized beta diversities within the same experiment are below (between − 11 and − 4) the − 1.96 standard deviation of the neutral distribution (sd). This indicates that beta diversity within experiments exhibited less dissimilarity than expected under the null hypothesis. The observed mean beta diversity between pairs of experiments at equivalent time was relatively high, with 87% (± 8%) of the compositional dissimilarities present (Fig. 3). Taxonomic diversity between these pairs was less than or equal to what randomization would predict. Four patterns of beta diversity dynamics can be identified:

First case: The observed beta diversity between pairs of experiments at equivalent times can be compared to the null model. For example, between “Experiment 1” and “Experiment 5”, the normalized beta-diversity was − 1.25 sd before H_2_ injection, -1.56 sd after injection, and − 0.18 sd at the furthest point. Similar dynamics were observed for “Experiment 5” compared to other experiments (pairs 1–5, 2–5, 3–5, and 4–5), as well as for pair 1–4.

Second case: Conversely, pairs 2–3 and 3–4 consistently showed less dissimilarity than predicted by neutral processes, with normalized beta diversity ranging from − 7.78 sd to -4,01 sd.

Third case: Pair 2–4 exhibited a unique pattern, starting with a null beta diversity of 0.09 sd before H_2_ injection, which changed to -1.30 sd after injection, ultimately converging to -3.58 sd at the furthest time point. At that stage, “Experiments 2, 3, and 4” shared a common community composed of ASVs related to OPB41 (1 ASV), *Desulfocucumis* (1 ASV), *Desulfolutivibrio* (1 ASV), *Acetobacterium* (1 ASV), *Rectinema* (6 ASVs), and *Tepidanaerobacter* (2 ASVs). Notably, ASVs related to *Rectinema* were also present in “Experiment 1”, and those related to *Acetabocterium* were also found in “Experiment 5”.

Fourth case: Pairs 1–2 and 1–3 had similar dissimilarities to the null model before injection. After H_2_ injection, their dissimilarities converged, and the signal disappeared at the furthest point. For pair 1–2, the dynamics started with a normalized dissimilarity of -0.67 sd; after injection, it decreased to -3.39 sd, then returned to an inconclusive value of -0.32 sd. Post-H_2_ injection, Experiments 1, 2 and 3 shared common ASVs from *Desulfocucumis* (6 ASVs) and *Rectinema* (5 ASVs), with some ASVs of *Rectinema* also present in “Experiment 4”. At the furthest time point, ASVs related to *Desulfocucumis* disappeared from Experiments 1 and 2 but remained in Experiment 3.

### Chord Diagram

**Fig. 4:**
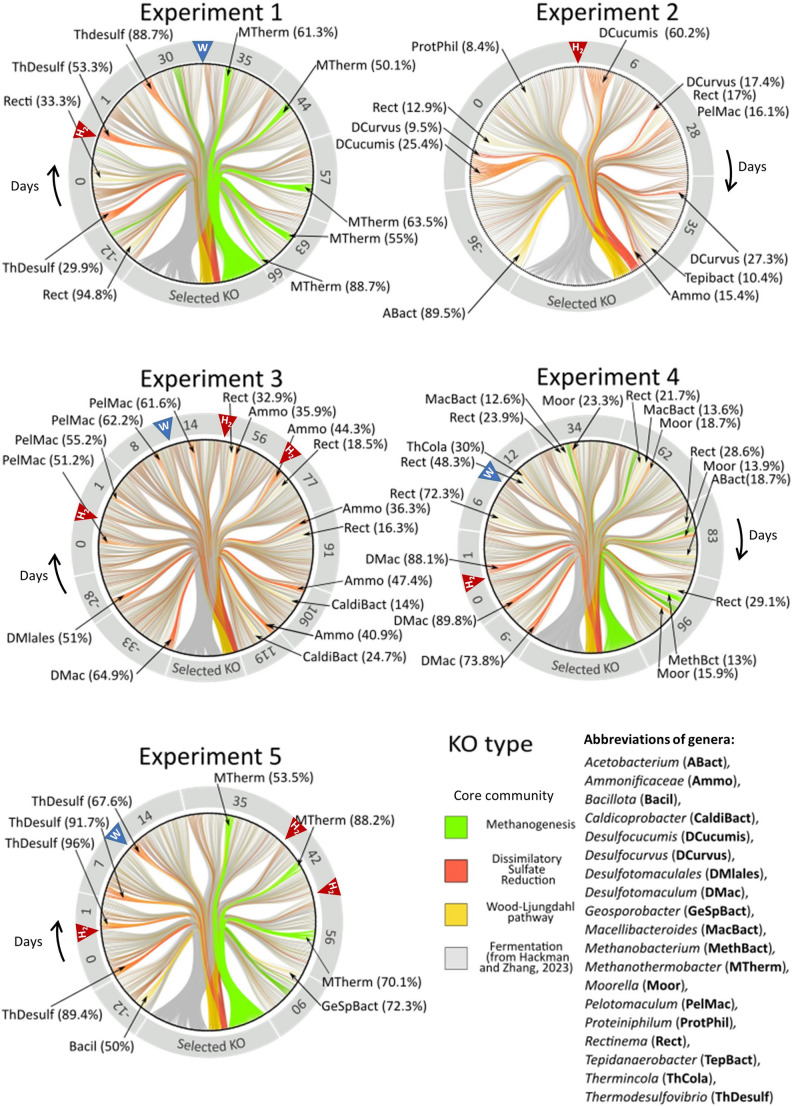
Distribution of major metabolic types based on cDNA data over time by experiment. Number in the crown repre sent days centered on H_2_ injection. The dial at the bottom represents KO nodes ordered by reference modules from KEGG for methanogenesis (M00567), dissimilatory sulfate reduction (M00596), and the Wood-Ljungdhal pathway (M00377). KO nodes related to fermentation where selected based on the work of Hackman and Zhang (2023). Other segments of the dial represent ASVs at the i^th^-sampling time. ASVs are clustered by genus affiliation. The colors of the edges represent the type of each KO attributed to a specific ASV: methanogenesis in green, dissimilatory sulfate reduction in red, the Wood-Ljungdhal pathway in yellow, and fermentation in grey. ASVs related to a genus or a group of genera are ordered by decreasing abundance or considered as the core community. The red triangle indicates H_2_ injection, and the blue triangle shows water refilling

The results of taxonomic diversity gathered from five experiments simulating H_2_ injection into deep aquifers used as geological storage for natural gas have been compiled into chord diagrams (Fig. 4). Based on their genetic sequence, each ASV was linked to selected functional genes related to methanogenesis, sulfate reduction, the Wood-Ljungdahl metabolism, and fermentation, using the PICRUSt method. Notably, fermentation emerged as the predictive metabolism process with the highest diversity of ASVs throughout the study, regardless of whether it was measured before or after H_2_ injection, and irrespective of its concentration (10% or 2%). Similarly, ASVs potentially capable of utilizing the Wood-Ljungdahl metabolic pathway were present across the study, although they were significantly fewer in number compared to those involved in fermentation. The biodiversity composition across all experiments showed that several ASVs (a total of 7) were shared among all incubations following the H_2_ injection. These ASVs were affiliated with *Acetobaterium* and were not detected during the H_2_ incubation phase. In addition to *Acetobacterium*, three other shared genera (*Proteiniphilum*, *Pseudomonas* and OPB41) were identified, although they were not represented by ASVs common to all experiments and were undetected during the incubation period. Despite this limited shared biodiversity, the major active ASVs belonged solely to 19 genera, including fermentative bacteria, methanogenic archaea, and sulfate-reducing bacteria. Functionally, it is noteworthy that the five microbial communities appeared to cluster into two main profiles: one comprising Experiments 1, 4, and 5, and the other consisting of Experiments 2 and 3.

In the first profile, sulfate-reducing bacteria were mainly observed during the early stages of the experiment, while methanogens began to dominate the community about a month after the initial (or sole) H_2_ injection. In Experiment 1, the two dominant genera before H_2_ injection (day 0) were the sulfate-reducing genus *Thermodesulfovibrio* (29.9% of total sequences) and the fermentative genus *Rectinema* (33.3%). By day 30 post-H_2_ injection, *Thermodesulfovibrio* comprised up to 88.7% of the sequences. At this point, ASVs belonging to the methanogenic genus *Methanothermobacter* began to proliferate, ultimately dominating the microbial community by day 66 after H_2_ injection (88.7%). In Experiment 4, *Rectinema* again appeared as the predominant fermentative bacterium (48.3% on day 12 after H_2_ injection), since sulfate was totally consumed 4 days after H_2_ injection. The sulfate-reducing genus *Desulfotomaculum* was dominant before H_2_ injection (89.8% on day 0), and although their activity decreased over time, they remained present as primary agents within this metabolic group. The diversity among methanogens was more limited, with all ASVs belonging to the genus *Methanobacter*, which never dominated the community, peaking at around 13%, while *Methanothermobacter* represented nearly 88% in Experiment 5 on the 66th day after H_2_ injection. Experiment 5 exhibited a similar succession of dominant genera as Experiment 1 after H_2_ injection, with *Thermodesulfovibrio* ranging from 67.6% to 96%, and *Methanothermobacter* from 53.5% to 88.2%. However, no representative of the genus Rectinema could be detected during the entire incubation. For these three communities, the injection of formation water on day 35 (Experiment 1), day 12 (Experiment 4), and day 14 (Experiment 5) did not revitalize sulfate reduction, even though sulfate was present in the water. Some differences were observed in Experiment 4, where initial sulfate reducers and methanogenic archaea were distinct, identified as *Desulfotomaculum* and *Methanobacterium*, respectively. Additionally, following the H_2_ injection, *Desulfotomaculum* disappeared due to rapid sulfate consumption, leading to a phase dominated by multiple fermentative genera such as *Rectinema*, *Moorella* or *Marcellibacteroides*. Experiments with Experiment 2 and Experiment 3 were characterized by the absence of methanogenic archaea, which were not detected even using qPCR, a more sensitive method (Mura et al. 2024a). Dissimilatory sulfate reduction continued until the end of the experiment. In Experiment 3, H_2_ did not trigger dissimilatory sulfate reduction, but water refill did in this experiment. Notably, in Experiments 2, 3, and 4, the communities were not dominated by a single genus, but by a core community, with *Rectinema* being a common fermentative genus during these phases.

## Discussion

In these studies, experiments simulated the sudden arrival of a large amount of H_2_into deep aquifer microbial communities (Haddad et al. 2022a; Mura et al. 2024a, b, 2025). These communities were developing under oligotrophic conditions with a redox potential below − 200 mV, favoring anaerobic prokaryotes. We report the subsequent variations in environmental conditions across four major temporal phases. The first phase is primarily characterized by a decrease in CO_2_, which can be attributed to both abiotic thermodynamic equilibrium phenomena between the gaseous and liquid phases, as well as biotic autotrophic processes (Fig. 1). Indeed, the microbial communities contain numerous fermentative bacteria belonging to genera such as *Caldicoprobacter*, *Rectinema*, and *Pelotomaculum*, which can thrive on microbial necromass and generate biological H_2_, supporting lithoautotrophic metabolism (Júnior et al. 2015; Imachi et al. 2007; Dong et al. 2018). In 2022, Haddad et al. (2022b) revealed that an intermittent production of H_2_ could be observed from a microbial community derived from the same site as Experiment 4 (Ab_L_1) before it was consumed by hydrogenotrophic sulfate-reducing bacteria. This H_2_ also supports other microorganisms, such as hydrogenotrophic methanogens, which, although below the detection threshold by high-throughput sequencing and even quantitative PCR approaches, are able to persist within the community. During this initial phase, an elution of ions such as fluoride and acetate from the rock can be observed, possibly originating from the drilling mud used during well construction (Mura et al. 2024b). Additionally, acetate is also a byproduct of the metabolism of diverse fermentative bacteria in these environments (e.g., *Acetobacterium* spp.), as well as from heterotrophic sulfate-reducing bacteria involved in the incomplete oxidation of organic molecules, including several species of the genus *Desulfotomaculum*, for example (Widdel and Back 2006; Fig. 4). An increase, and even a long plateau, in acetate does not mean it is not being consumed; if it is, the consumption is minimal, except in Experiment 3. In Experiment 3, the peak of acetate at the start of incubation was almost completely consumed before H_2_ injection. Following H_2_ injection, this consumption capability seems to have diminished, as a new accumulation of acetate was subsequently recorded. In light of the overall results, and although it is impossible at this stage to provide a definitive explanation, the hypothesis of a decrease, or even a cessation, of acetoclastic metabolism following H_2_ injection (from 0.75 mmol H_2_∙kg of water^− 1^ to 6.66 mmol H_2_∙kg of water^− 1^) is proposed.

In the second phase, H_2_ is the most variable parameter, influenced by its H_2_ injection rates of 2% and 10% depending on the experiments. The reduction of CO_2_ intensifies with increased lithoautotrophic metabolism, particularly among sulfate-reducing functional groups. Taxonomically, sulfate-reducers vary among the deep aquifers studied: *Desulfocucumis* for Aquifers 1, 2, and 3; *Desulfofarcimen* for Aquifers 3 and 4; *Desulfotomaculum* for Aquifers 3, 4, and 5; *Ammonificaceae* for Aquifers 3 and 5; and *Thermodesulfovibrio* for Aquifers 1 and 5. At the same time, experiments on Experiments 1, 2, 4, and 5 demonstrated the production and accumulation of formate. This formate production is clearly linked to lithoautotrophic metabolism (Haddad et al. 2022a, b). It has been hypothesized that homoacetogenesis may be halted at the first step due to energetic constraints caused by cellular cytoplasmic acidification from high CO_2_ partial pressure (Stoll et al. 2018). Furthermore, it is known that anaerobic microorganisms capable of utilizing formate and H_2_/CO_2_ (such as sulfate-reducers, homoacetogens, and methanogens) can produce substantial amounts of formate when grown in high concentration of H_2_/CO_2_ (Peters et al. 1999). In this context, formate serves as an intermediate whose concentration increases alongside rising H_2_ levels, until it leaks out of the cell. Such formate production has been observed in anoxic environments rich in H_2_ (Bleicher and Winter 1994; Lajoie et al. 1988; DeGraaf and Cappenberg 1996). Notably, the sulfate-reducing Firmicute *Desulfotomaculum orientis* has demonstrated this capability (Peters et al. 1999).

The third phase is characterized by an increase in formate. Formate concentrations reached approximately 6.6 mM for Experiment 1, 2.5 mM for Experiment 2, 0.3 mM for Experiment 3, 3.3 mM for Experiment 4, and 5.5 mM for Experiment 5 (Mura et al. 2024a, b, 2025). In the experiments involving Aquifers 1, 4, and 5, sulfate was rapidly depleted following H_2_ injection, well before formate concentrations peaked. Conversely, in Experiments 2 and 3, the initial sulfate concentrations in formation water were higher than in the other aquifers (Aquifer 2: 13.03 mM; Aquifer 3: 25.06 mM). In Experiment 3, with no formate detected, sulfate reduction continued until the end of the experiment. In Experiment 2, with formate present, sulfate reduction and lithoautotrophy slowed dramatically, nearly ceasing despite excess sulfate, H_2_, and CO_2_. In 2022, Voskuhl and colleagues demonstrated in laboratory experiments using model sulfate-reducing strains that high formate concentrations (8 mM) could inhibit sulfate reduction. They also noted that such inhibition might occur at lower concentrations (approximately 2.37 mM) under in situ conditions. To explain the differences between laboratory cultures and deep environments, the authors suggested that harsher conditions could impose additional stress. It is also important to note that they did not test intermediate concentrations between 2.5 mM and 8 mM, leaving open the possibility that inhibition occurs at concentrations below 8 mM, even in laboratory settings. In this context, it is plausible that the inhibition of sulfate reduction observed in the Experiment 2 is related to formate accumulation due to its production following H_2_ injection. This third phase is also marked by decreases in sodium, potassium, chloride, and calcium, ions that can precipitate as carbonate minerals as alkalinity increases (Haddad et al. 2022a).

Finally, the fourth phase presents the lowest sulfate concentrations, which are entirely depleted. In the case of digesters, high H_2_ concentrations have been shown to inhibit fermentative bacteria that produce it (Ding and Zhao 2018; Perret et al. 2024). The significant decline in active richness observed in Experiment 2 clearly reflected by a loss of 36 ASVs, mainly affiliated with fermentative organisms such as *Anaerovorax* or *Macellibacteroides*. A similar pattern appears in Experiments 1, 3, and 5, where fermentative bacteria are rapidly lost following H_2_ injection. These losses are substantial enough to cause temporary taxonomic convergence between Experiments 1 and 2, and between Experiments 1 and 3, after H_2_ injection. In other words, certain fermentative species are excluded post-injection, reducing overall richness and increasing the proportion of shared communities across these experiments. Despite this selective suppression, some fermentative ASVs such as those related to *Rectinema*, *Caldicoprobacter*,* and Pelotomaculum*, persisted at three tested time points. However, some ASV associated with these conserved genera over time in the same experience may be lost. This phenomenon could indicate possible ecotype selection or an artifact resulting from PCR amplification. These ASVs belong to genera represented by multiple ASVs, such as *Calicroprobacter*, which is found in Experiment 1 (5 ASVs), Experiment 2 (7 ASVs), Experiment 3 (5 ASVs), and Experiment 5 (5 ASVs). For instance, H_2_ has also been found to slow down or even inhibit growth rates of members of the genus *Thermococcus*, although this effect can be mitigated if organisms can utilize formate for reducing power elimination (Topçuoğlu et al. 2018).

The deep aquifers targeted in this study, ranging from 582 to 1,185 m AMSL, lack most electron acceptors found in surface environments, including anoxic ones. In these deep, nutrient-poor environments, the primary electron acceptors are sulfate and CO_2_. In UGS sites, the main carbon source is the natural gas stored, where some hydrocarbons can diffuse into the formation water, nourishing anaerobic heterotrophic bacteria (Aüllo et al. 2016; Ranchou-Peyruse et al. 2019, 2021a, b). Over 25 years, the gas operator observed at the Ab_L_1 site, for example, a constant decrease in sulfate at the gas-water interface of the aquifer (Ranchou-Peyruse et al. 2019), with sulfate-reducers consuming sulfate faster than it could be replenished by the movement of formation water. The low in situ sulfate concentration in Experiment 1 suggests a similar phenomenon might be occurring. Although iron-oxidized minerals (hematite, goethite) can be found in some of these rocks (not detected in the rocks used for the experiments), particularly in the Triassic layer, their low concentration likely doesn’t support ferric-reducing organisms in such a reduced environment where ferrous (Fe^2+^) ions are more prevalent than ferric (Fe^3+^) ions (Bagnoud et al. 2016).

In ecosystems with limited diversity of terminal electron acceptors, fermentative bacteria comprise most of the taxonomic diversity. Importantly, their significant presence at the start of the experiment is likely due to the waiting period between sampling and incubation in the high-pressure reactor (Ranchou-Peyruse et al. 2023). To counteract this, communities are initially kept in the high-pressure reactor under conditions simulating current UGS scenarios to restructure them to a state closer to their original environment.

Given that these deep microbial communities are raltively simplified in terms of possible energetic metabolisms, it allows for the reliable utilization of results obtained from *16 S rRNA* gene-based taxonomic diversity analyses. Indeed, sulfate reduction and methanogenesis are carried out by phylogenetically well-separated microorganisms, and their activities could be well correlated with the evolution of sulfate, H_2_, CO_2_, and carbonates, in particular. The identification of fermentative, acetogenic and formatogenic bacteria is a bit more speculative, because while their presence is unquestionable, their identification based on 16 S rDNA is often hypothetical. All detected ASVs can be associated with one or more metabolic pathways (selected KO): fermentation, sulfate reduction, the Ljundahl-Wood pathway, and methanogenesis. In Experiments 1, 2, 4, and 5, sulfate reduction was preserved and enhanced after H_2_ injection. In the case of Experiment 3, sulfate reduction is not triggered by the first hydrogen pulse. In Experiment 2, some ASVs have been associated with a representative of the genus *Desulfocurvus*, which was previously isolated from a deep aquifer UGS (Klouche et al. 2009). Members of this genus are described as utilizing formate in sulfate reduction, and some are capable of using H_2_ in the presence of acetate as a carbon source (Hamdi et al. 2013). In Experiment 3, the ASVs affiliated with the family *Ammonificaceae* (Mura et al., 2024) are closely related to the sulfate-reducing strain KNH (AB518055; 99% identity with the partial *16 S rRNA* sequence [377 nt]) isolated from saline water at a depth of 1,000 m, which is similar to *Desulfotomaculum kuznetsovii* and *Desulforudis audaxviator* (Nakamura et al. 2011).

Sulfate depletion provides an initial categorization, distinguishing between groups where sulfate is not fully consumed (Experiments 2 and 3) and those in which sulfate is completely depleted, followed by a phase of methanogenesis (Experiments 1, 4, and 5). While sulfate concentration is undoubtedly an important environmental parameter, it would be overly simplistic to view it as the sole environmental driver of the microbial taxonomic diversity of the community. Although Experiments 1 and 4 feature low sulfate concentrations (14 mg∙L^− 1^ and 9 mg∙L^− 1^, respectively), Experiments 2 and 3 have much higher levels (1,252 mg∙L^− 1^ and 2,407 mg∙L^− 1^), and Experiment 5 initially has a relatively high concentration (951 mg∙L^− 1^). However, complete depletion occurs within just 45 days (Mura et al. 2025), allowing the development of *Methanobacter wolfeii*, whose ASVs represent 77.3% of the total sequence. The principal archaeal composition further distinguishes Experiments 1 and 5, which are dominated solely by *Methanothermobacter*, and Experiment 4, where *Methanobacterium* is the predominant methanogenic archaeon. By the end of the incubation period, ASVs of *Methanothermobacter* are detected.

However, the categorization of experiments’ chemical dynamic established above is not supported by beta diversity analyses, which show that Experiments 1 and 5 do not share significant common biodiversity. Specifically, these aquifers share five to six ASVs related to *Thermodesulfovibrio-*affiliated ASV, and the normalized taxonomic beta-diversity is insufficient to categorize them as convergent communities. In contrast, pairs of Experiments 2 and 3, and pairs of Experiments 3 and 4, exhibit shared biodiversity. This convergence increases since Experiments 2, 3, and 4 show significant shared biodiversity at the “furthest” sampling times, 28 days, 8 days, and 6 days after H_2_ injection, respectively, though they do not exhibit the same dominance or metabolic trajectory over time. These observations reveal a disconnection between taxonomic diversity and community metabolic functioning in the aquifers, challenging the ability to predict dominance based on previous samples. This disconnection is further evidenced by the high dissimilarity observed between randomly paired experimental samples, which show approximately 90% Jaccard dissimilarity in presence-absence data, suggesting potential dispersal limitations already observed in community aquifer assembly (Beaton et al. 2016; Putman ). Moreover, major metabolisms in the communities are capable of fixing carbon dioxide and available carbonate, causing an alkalinization of the environment (Mura et al. 2025; Shojaee et al. 2024). In the case of Experiments 2, 3, and 4, alkalinisation remains circumneutral or slightly basic, whereas in Experiments 1 and 5, alkalinisation is more pronounced; in Experiment 5, the modeled pH could reach as high as 10 during methanogenesis. Alkalinization is a well-know selective pressure (Merino, 2019) and may influence the community structuring in Experiments 1 and 5. Interestingly, the presence of dispersal limitations and the selective pressure associated with pH are also evident in serpentinization systems (Putman et al. 2021).

UGS facilities utilize porous reservoirs such as depleted hydrocarbon reservoirs and deep aquifers to store natural gas. These facilities undergo cyclic gas injection and withdrawal, which alternately increase and decrease the storage volume, impacting the displacement of formation water. This phenomenon is often referred to as the “breathing” of the storage. When far from the storage site, the displacement of formation water is estimated to be slow (Ranchou-Peyruse et al. 2019), but closer to the storage site, this rate can be accelerated. Nonetheless, the maximum storage volume remains constant, dictated by regulatory considerations. This confinement of the gas/water interface, where most exchanges between the two phases occur, can lead over time to significant depletions of certain nutrients or electron acceptors (Ranchou-Peyruse et al. 2019). Consequently, it is plausible to observe these two scenarios of microbial community dynamics within these areas characterized by the gas/water interfaces. In laboratory conditions, the arrival of nutrient-rich water was simulated by injecting formation water filter-sterilized through a 0.1 μm filter. This influx of nutrients resulted in changes in alpha diversity within the active microbial communities (cDNA) and, in some cases, significantly disrupted existing metabolic functions following water refilling. In Experiment 1, water injection occurred relatively close to the threshold between sulfate reduction and methanogenesis (Experiment 1, between days 30 and 35). Although sulfate depletion marks a shift in dominant metabolisms of primary producers, we hypothesize that water injection may have disturbed the community and accelerated the transition from sulfate reduction to methanogenesis. Conversely, in Experiment 3, water injection triggered sulfate reduction by introducing new nutrients. In Experiments 4 and 5, no significant disruption in metabolic diversity was observed (Fig. 4). This suggest a potential depletion of sulfate in these zones. The absence of microorganisms capable of consuming formate in the presence of H_2_ and CO_2_ could lead to local accumulations of formate, which might inhibit sulfate reduction (Voskuhl et al. 2022). Additionally, lithoautotrophy at the gas-water interfaces causes alkalinization of the formation water, potentially leading to carbonate mineral precipitation (Berta et al. 2018; Dopffel et al., ; Fournier et al. 2023; Ranchou-Peyruse et al. 2024). This process decreases available CO_2_ and carbonate ions for autotrophic organisms (Mura et al. 2025), while the rising pH imposes stress on the microbial community and favors microorganisms adapted to such conditions. Unlike surface ecosystems continuously replenished by new microorganisms transported by wind, meteoric water and aquatic currents, the microbial ecosystems at the edges of UGS facilities likely face severe biomass reduction and increased competition for inorganic carbon sources. This scenario may also involve cooperative strategies such as syntrophic lifestyles or metabolic versatility to survive under harsh conditions. Additionally, the setup does not consider aquifer heterogeneity, which could influence microbial community responses to H_2_ in promoting different community compositions and functions.

In summary, community incubations show that, in most cases, fermentative species are inhibited immediately after H_2_ injection. These phenomena could be expected after injecting H_2_ into UGS. Taxonomic biodiversity in the aquifer seems clearly influenced by dispersal limitation, making it difficult to predict major dominance based on biodiversity. However, this work highlights the emerging successional dominance of *Thermodesulfovibrio* and *Methanothermobacter* as efficient H_2_ scavengers. This could reflect the possibility of a succession of dominant species under harsh conditions and suggest a certain predictability. Water refills seems to have a moderate impact on the community, sometimes disrupting the metabolic functioning in the simulated ecosystem. This disruption raises questions about the stability of microbial ecosystem functioning within deep aquifers used as UGS. It is important to note that this study only utilizes five experimental simulations of H_2_ injection, and that further are still necessary, particularly to better highlight key nutrients and the potential effect of different species within the same functional group. Nevertheless, by observing the evolution of microbial community structuring at the metabolic level (fermentation, sulfate reduction, acetogenesis, methanogenesis), two evolutionary scenarios appear to emerge, which could be exploited in future biogeomodelling efforts aimed at simulating the evolution of future H_2_ storage.

## Supplementary Information

Below is the link to the electronic supplementary material.

Supplementary Material 1

## Data Availability

No datasets were generated or analysed during the current study.
