# Supplementary material for: Successional Trajectories of Deep Subsurface Microbiomes in Response To Experimental Dihydrogen Injection
Source: Microb Ecol. 2026 Feb 11;89(1):60. doi: 10.1007/s00248-026-02697-3 (PMC12948927; doi:10.1007/s00248-026-02697-3)
Supplement: Supplementary file 1 — Supplementary Material 1 [file 248_2026_2697_MOESM1_ESM.docx]

SUPPLEMENTARY MATERIAL:

**Disconnection between physical chemistry dynamics and biodiversity during experimental dihydrogen pulse**

Authors : *Antoine Lafont^1^, Cyrille Violle^2*^, Magali Ranchou-Peyruse^1,3^, Marion Guignard^1^, Jean Mura^3^, Tiphaine Fargetton^4^, Pierre Cézac^3^ & Anthony Ranchou-Peyruse^1*^*

^1^ Universite de Pau et des Pays de l’Adour, E2S UPPA, CNRS, IPREM, Pau, France

^2^ CEFE, Univ Montpellier, CNRS, EPHE, IRD, Montpellier, France

^3^ Universite de Pau et des Pays de l’Adour, E2S UPPA, LaTEP, Pau, France

^4^ Storengy, Bois-Colombes, ENGIE La Défense, France

* Corresponding authors: cyrille.violle@cefe.cnrs.fr and anthony.ranchou-peyruse@univ-pau.fr

List of content

**Table S1 : Physicochemical properties of the sampled formation water.**

**Figure S1 : Temporal response of deep gas storage microbial communities’ alpha diversity to experimental hydrogen pulse.** Columns segregated each community, from Experiment 1 to 5, rows indicate to alpha diversities metrics, Shannon (1948) index ans Stoddart index (1983), and the index partitions, richness and evenness estimated with Pielou’s metric (Pielou, 1966). Alpha diversity were estimated on ASV. Red and blue line indicated if each metrics were estimated on total community (DNA asv) or active one (RNA community).

**Figure S2: Time heterogeneity between pairs does not influence the results.** On the y-axis, normalized beta diversity by a null hypothesis; on the x-axis, the time difference between experiments. Spearman correlation between time differences and centered beta diversity for the entire dataset (ρ = -0.077, p = 0.687), the "After" group (ρ = -0.213, p = 0.554), and the "Furthest" group (ρ = 0.294, p = 0.410) are not significant.

**Table S1 : Physicochemical properties of the sampled formation water.**

|  | **Parameters** |  | **Value** |  |  |  | **Unit** |
| --- | --- | --- | --- | --- | --- | --- | --- |
| **Site characteristics** | Site | Pb_T_1 | Pb_J_11 | Pb_C_5 | Ab_L_1 | Pb_T_5 |  |
|  | Depth | 989 | 836 | 840 | 505 | 1195 | m |
|  | Geological formation | Triassic | Sequanian | Neocomian | Eocene/Lutetian | Triassic |  |
|  | Location | Parisian basin | Parisian Basin | Parisian basin | Aquitaine basin | Parisian basin |  |
|  | Pressure | 95 | 85 | 85 | 60 | 115 | Bar |
|  | Temperature | 47 | 35 | 41 | 23.4 | 53 | °C |
|  | pH | 7.9 | 8.0 | 8.5 | 7.7 | 8.0 |  |
|  | Redox Potential | -365.6 | -298.6 | -178 | -283 | 49 | mV |
|  | Conductivity at 25°C | 1.2 | 6.5 | 6.2 | 0.3 | 6.5 | mS/cm |
|  | Organic Carbon | <1 | <1 | <1 | <0.3 |  | mg/L |
| **Water composition** | Chloride | 7.1 | 11.62 | 8.04 | 0.2 | 39.43 | mM |
|  | Nitrate | <0.0016 | <0.0016 | <0.0016 | 0.0001 | <0.0016 | mM |
|  | Nitrite | <0.0004 | <0.02 | <0.02 | <0.0003 | <0.0004 | mM |
|  | Sulfate | 0.15 | 13.03 | 25.06 | 0.03 | 9.90 | mM |
|  | Carbonate | <1 | <1 | <1 | <0.0083 | <1.00 | mM |
|  | Bicarbonate | 8.51 | 34.45 | 12.31 | 3.21 | 12.55 | mM |
|  | Calcium | 0.26 | 1.24 | 0.63 | 1.26 | 1.12 | mM |
|  | Ferrous iron | <0.89 | 0.08 | 0.21 | 4.57 |  | µM |
|  | Total iron | 6.88 |  | 0.40 |  | 2.54 | µM |
|  | Magnesium | 0.14 | 1.57 | 0.83 | 0.26 | 0.78 | mM |
|  | Potassium | 0.27 | 0.65 | 0.82 | 0.16 | 0.68 | mM |
|  | Sodium | 14.89 | 62.782 | 65.35 | 0.52 | 58.29 | mM |
|  | Reference | Haddad et al., 2022 | Mura et al., 2024 | Mura et al., 2024a | Mura et al.,  2024b | Mura et al.,  2025 |  |

**Figure S1: Temporal response of deep gas storage microbial communities’ alpha diversity to an experimental hydrogen pulse.** Columns represent individual communities, from Experiment 1 to 5. Rows indicate alpha diversity metrics: the Shannon (1948) index and the Stoddart (1983) index, along with their partitions, richness and evenness, as estimated by Pielou’s metric (Pielou, 1966). Alpha diversity was calculated on ASV data. The red and blue lines indicate whether each metric was estimated on the total community (DNA) or the active community (RNA).


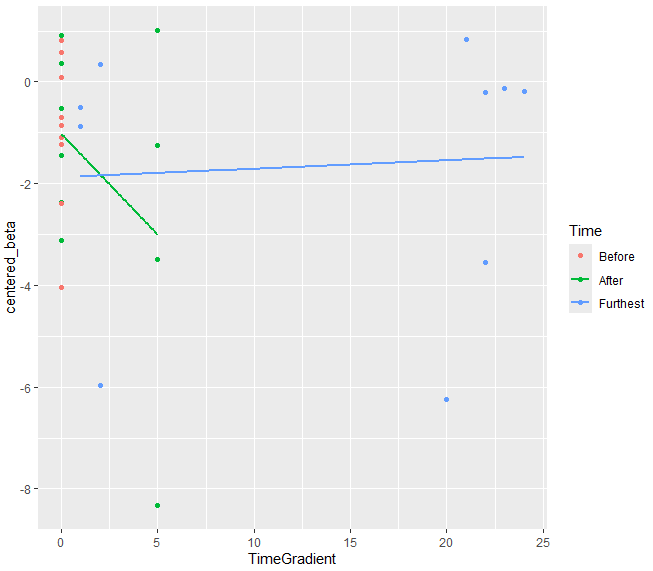


**Figure S2: Time heterogeneity between pairs does not influence the results.** On the y-axis, normalized beta diversity by a null hypothesis; on the x-axis, the time difference between experiments. Spearman correlation between time differences and centered beta diversity for the entire dataset (ρ = -0.077, p = 0.687), the "After" group (ρ = -0.213, p = 0.554), and the "Furthest" group (ρ = 0.294, p = 0.410) are not significant.

References:

Pielou, E. C. The Measurement of Diversity in Different Types of Biological Colledions. Journal of Theoretical Biology **1966**, 13,131-144.

Shannon C. E. A Mathematical Theory of Communication, The Bell System Technical Journal **1948**, 27(3) 380-423.

Stoddart, J. A. A Genotypic Diversity Measure. *Journal of Heredity* **1983**, *74* (6), 489–490.
